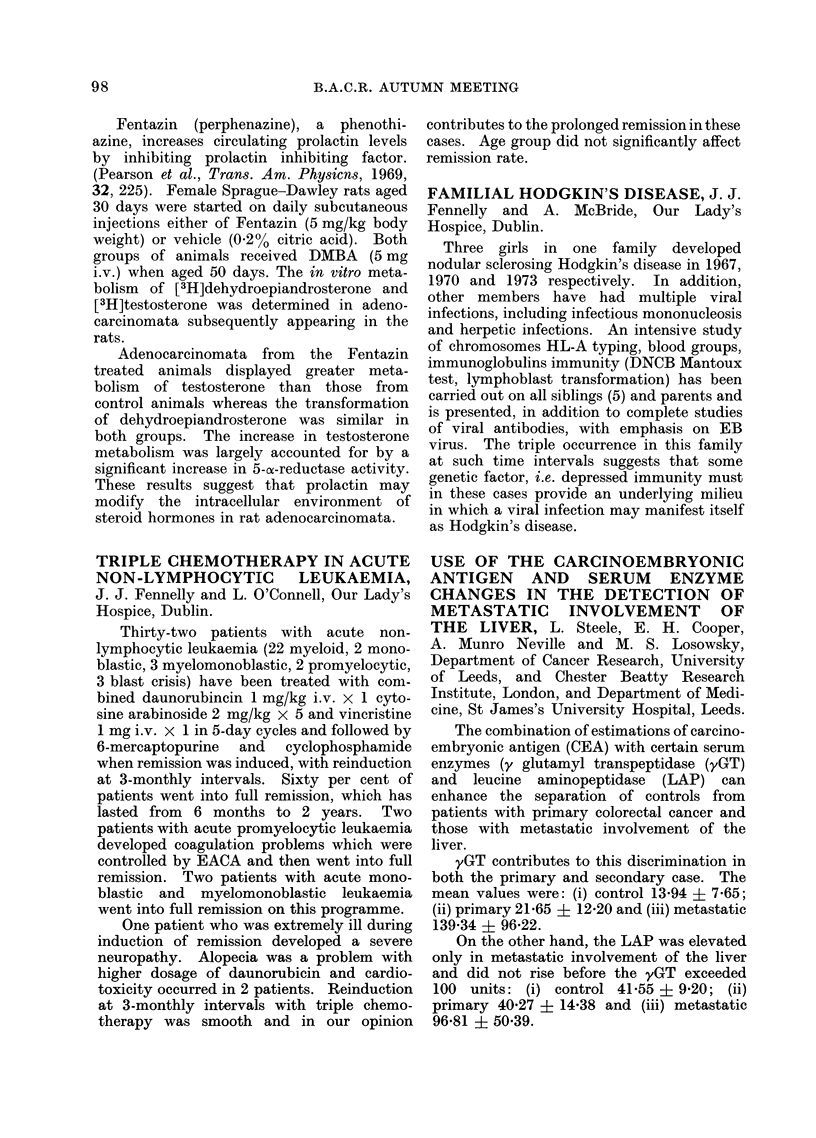# Proceedings: Familial Hodgkin's disease.

**DOI:** 10.1038/bjc.1974.35

**Published:** 1974-01

**Authors:** A. J. Fennelly, A. McBride


**FAMILIAL HODGKIN'S DISEASE,** J. J. Fennelly and A. McBride, Our Lady's Hospice, Dublin.

Three girls in one family developed nodular sclerosing Hodgkin's disease in 1967, 1970 and 1973 respectively. In addition, other members have had multiple viral infections, including infectious mononucleosis and herpetic infections. An intensive study of chromosomes HL-A typing, blood groups, immunoglobulins immunity (DNCB Mantoux test, lymphoblast transformation) has been carried out on all siblings (5) and parents and is presented, in addition to complete studies of viral antibodies, with emphasis on EB virus. The triple occurrence in this family at such time intervals suggests that some genetic factor, *i.e.* depressed immunity must in these cases provide an underlying milieu in which a viral infection may manifest itself as Hodgkin's disease.